# 

**Published:** 1896-02-29

**Authors:** 


### CONTENTS.

**The Factors of National Health** - - - - - - - 355
**Sunshine on the South Coast** - - - - - - - - 356
**Dyspepsia and Consumption.** By SOLOMON C. SMITH, M.D.
II.—The Forms of Dyspepsia - - - - - - - - 357
**The Ophthalmology of the Poets.—II.** - - - - - 358
**Annotations.**—A Cruel "Surgical Operation"—Sunshine, Air, and Germs—An Injudicious Definition - - - - - - 359
**Notes and News** - - - - - - - - - - 360
**Editor's Letter Box** - - - - - - - - - 370
**The Book World of Medicine and Science** - - - - 371
**The Student of Medicine** - - - - - - - - 372
**Medical Progress and Hospital Clinics—**
Acute Necrosis. By E. PERCY PATON, M.D. - - - - - 361
PROGRESS IN MEDICINE.—Supra-Renal Bodies - - - - 363
PROGRESS IN SURGERY.—Hernia - - - - - - - 363
NEW APPLIANCES AND THINGS MEDICAL - - - - - - 366
**The Institutional Workshop—**
THE OUT-PATIENT SYSTEM AND ITS ABUSE - - - - - 367
HOSPITAL CONSTRUCTION - - - - - - - - 368
THE STORY OF THE INSANE FROM YEAR TO YEAR - - - 368
HOSPITAL MEETINGS - - - - - - - - - - 369
PRACTICAL DEPARTMENTS - - - - - - - - 370

### EXTRA SUPPLEMENT.—THE NURSING MIRROR.

**News from the Nursing World.**—New Home for Nurses at St. Thomas's Hospital—The Chertsey Board of Guardians—Medical Women in Scotland—An Example to Cottage Hospitals—Nurses' Home, Dundee—The Sarah Acland Home—District Nurses at Ryde—District Nursing in Birmingham—District Nursing in Holland—Explosion at Johannesburg — Midwife and Nurse — Probationers Wanted—Short Items - - - - - - - - clxxxvii

**Lectures on Nursing.** XI.—Typhoid Fever - - - - clxxxix

**Workhouse Infirmary Nursing** - - - - - - - cxc

**Pirated Advertisements** - - - - - - - - cxc

**Opening of Wards at St. Thomas's Hospital** - - - cxci

**Royal National Pension Fund for Nurses.**—The Nurses' Gift to H.R.H. the Princess Maud of Wales - cxci

**On Certain Aspects of the Nursing Question as seen in England and Germany.—II.** - - - - cxcii

**Some Experiences of a Lecturer on Sanitation** - - cxciii

**Talks about Food** - - - - - - - - - - cxciv

**Everybody's Opinion** - - - - - - - - - cxcv

**Practical Points** - - - - - - - - - - cxcv

**Wants and Workers** - - - - - - - - - cxcv

**The Book World for Women and Nurses** - - - - cxcvi

**Notes and Queries** - - - - - - - - - - cxcvi

**For Reading to the Sick** - - - - - - - - cxcvi

